# Trigonelline, An Alkaloid From *Leonurus japonicus* Houtt., Suppresses Mast Cell Activation and OVA-Induced Allergic Asthma

**DOI:** 10.3389/fphar.2021.687970

**Published:** 2021-08-04

**Authors:** Wenhui Zhang, Yingling Zhang, Simin Chen, Hong Zhang, Man Yuan, Lianbo Xiao, Yue Lu, Hongxi Xu

**Affiliations:** ^1^ School of Pharmacy, Shanghai University of Traditional Chinese Medicine, Shanghai, China; ^2^ Institute of Arthritis Research, Shanghai Academy of Chinese Medical Sciences, Guanghua Integrative Medicine Hospital, Shanghai, China; ^3^ Shuguang Hospital, Shanghai University of Traditional Chinese Medicine, Shanghai, China

**Keywords:** *Leonurus japonicus* Houtt, trigonelline, mast cells, inflammatory, allergic asthma

## Abstract

Trigonelline, one of the active compounds from *Leonurus japonicus* Houtt., has been proven to have pharmacological value in diabetes, the central nervous system and cardiovascular diseases. Recent studies have shown that it may also be beneficial in controlling inflammation. However, the mechanism of the antiallergic effects of trigonelline has not been well studied. As the key effector cells participating in the development of allergies, mast cells have been linked to the pathogenesis of asthma for ages. In this study, we demonstrated the inhibitory effect of trigonelline on activated bone marrow-derived mast cells (BMMCs) and verified its anti-inflammatory properties using an ovalbumin (OVA)-induced asthma model. Trigonelline suppressed BMMC degranulation and decreased the production of the cytokines, prostaglandin D_2_ (PGD_2_) and leukotriene C_4_ (LTC_4_) in a dose-dependent manner. The potent mechanism is mainly through the suppression of the nuclear factor kappa B (NF-κB) and mitogen-activated protein kinase (MAPK) signaling pathways. Trigonelline can alleviate pathological damage in lung tissue and reduce the levels of serum immunoglobulin E (IgE) and T helper 2 (Th2) cytokines. RNA-seq results revealed the HIF-1α to be a potential target for the allergic reaction. Taken together, our study demonstrated that trigonelline can inhibit allergic inflammation *in vitro* and *in vivo*, which may provide a basis for novel anti-inflammatory drug development.

## Introduction

Allergic asthma is a worldwide disease characterized by local airway inflammation and hyperresponsiveness, followed by the activation of the immune system and the release of multiple cytokines (Mims, 2015). Inhaled corticosteroids alone or in combination with long-acting bronchodilators are the current therapeutic asthma strategies (Finotto, 2019), but the side effects behind these therapies have not yet been addressed. Currently, the lack of treatment options is a serious challenge for allergic asthma.

Mast cells, also known as immediate hypersensitivity effector cells, are abundantly found in barrier tissues in contact with the external environment, such as the skin or the mucosa of the respiratory and gastrointestinal tracts (Saito et al., 2013). Mast cells play a prominent role in promoting various chronic inflammatory disorders such as asthma, allergic rhinitis, anaphylaxis and rheumatic disease (Méndez-Enríquez and Hallgren, 2019). Mast cells can be stimulated through many kinds of surface receptors, including the high-affinity receptor for IgE (FcεRI), receptor for stem cell factor (c-Kit), cytokine receptors, thus releasing three main classes of mediators: preformed granule-associated mediators histamine, serotonin and tryptase); newly generated lipid mediators (PGD_2_, LTC_4_); and various cytokines and chemokines such as TNF-α, IL-1β, IL-6, IL-13, CCL5 and CCL8 (Moon et al., 2014). Therefore, mast cells seem to be a critical target in allergy-related diseases.

*Leonurus japonicus* Houtt. is a traditional Chinese medicine that functions in promoting blood circulation, regulating menstruation, clearing heat and detoxification (Shang et al., 2014). The alkaloids in *Leonurus japonicus* Houtt. have roles in anti-inflammation and immune regulation (Shin et al., 2009). Several studies have demonstrated that its anti-inflammatory effects are mainly related to gynecological diseases such as endometritis and mastitis. Apart from its traditional medicinal value, we hope to find a new use in inflammatory diseases. Trigonelline, one of the total alkaloids from *Leonurus japonicus* Houtt., can also be found in coffee and fenugreek. Trigonelline can have partial therapeutic effects to attenuate the cardiac manifestations of colitis (Omidi-Ardali et al., 2019). Previous research also showed that trigonelline has anti-degranulation properties (Nugrahini et al., 2020), and *Trigonella foenum-graecum* L. extracts cure Th2-induced allergic skin inflammation by enhancing Th1 differentiation (Bae et al., 2012). However, the mechanism of the antiallergic effects of trigonelline has not been well studied. In this study, we investigated the effect of trigonelline hydrochloride (TH) on IgE/Ag-activated BMMCs and elucidated the possible mechanism for its inhibitory effect through passive systemic anaphylaxis (PSA) and an OVA-induced asthma model *in vivo*.

## Materials and Methods

### Reagents

OVA, DNP-IgE, and DNP-HSA were purchased from Sigma Aldrich (MO, United States), with a purity of ≥90.0%. RMPI-1640, fetal bovine serum (FBS), penicillin, and streptomycin were purchased from Gibco (Grand Island, NY/Carlsbad, CA, United States). 2,5-Diphenyltetrazolium bromide (MTT), and DNP-HSA were obtained from Sigma Chemical Co. (St.Louis, MO, United States). Milli-Q water was supplied from a water purification system (Millipore, MA, United States). Horseradish peroxidase (HRP)-conjugated goat anti-rabbit IgG was purchased from Invitrogen (Carlsbad, CA, United States), and Alum Adjuvant (Thermo Scientific, United States). Trigonelline hydrochloride (Sigma-Aldrich, Buchs, Switzerland; -analytical standard) was dissolved in PBS and adjusted to the required concentration.

### Culturing of Bone Marrow Derived Mast cells

Female BALB/c mice (5–6 weeks) were sacrificed and hind legs were dissected. Bones were separated and flushed with serum-free RPMI-1640. 35% Pokeweed Mitogen-Spleen Cell Conditioned Medium (PWM-SCM) was used to culture bone marrow cells. The medium was changed when the cell density reached 70–80%. After 5–6 weeks BMMCs were mature and flow cytometry was performed to confirm according to a previously described protocol (Nagashima et al., 2019).

### MTT Assay for Cell Viability

Cytotoxicity studies were performed using the MTT assay. BMMCs (1 × 10^5^ cells/ml )were incubated for 4 h in the presence of TH (0.01–1 mM) in 96-well plates. Then, 0.5 mg/ml of MTT was added. After an additional 4 h incubation at 37°C, the medium was discarded and 150 μl of 0.04 N HCl/isopropanol was added to each well. Optical density was measured at 570 nm.

### β- Hexosaminidase Release Assay

BMMCs were pretreated with DNP-IgE (500 ng/ml) in 1640 medium for at least 2 h. Cells diluted to 1 × 10^6^ cells/ml were seeded in 96 well plates and treated with or without TH for 1 h before stimulation with 100 ng/ml DNP-HSA for 15 min. After harvesting the supernatant, 50 μl of β-hex substrate solution (1.3 mg/ml p-nitrophenyl-2-acetamido-2-deoxy-β-D-glucopyranoside in 100 mM sodium citrate, pH 4.5) was added to each well of 96-well plates and then incubated at 37°C for 60 min. The reaction was stopped by adding 175 μl of 0.2 M glycine-NaOH (pH 10.7). The absorbance at 405 nm was measured in a microplate reader. The percentage of β-hex released into the supernatant was calculated by the following formula: [S%(S + P)] × 100, where S and P are the β-hex contents of the supernatant and cell pellet, respectively.

### Measurement of Inflammatory Cytokines

BMMCs were pretreated with DNP-IgE (500 ng/ml) in RPMI-1640 medium for at least 2 h before performing assays. BMMCs (1 × 10^6^ cells/ml) were incubated for 1 h in the presence of TH (0.1 mM and 0.2 mM) in 12-well plates. Then, the cells were stimulated with DNP-HSA for 6 h at 37°C and 5% CO_2_ in an incubator. Cell suspensions were collected and centrifuged at 10000 rpm for 3 min at 4°C. According to the manufacturer’s instructions, supernatants were used for analysis of IL-6 and TNF-α concentrations using ELISA assays (R&D Systems, Minneapolis, MN, United States).

### Measurement of LTC_4_ and PGD_2_

BMMCs were pretreated with DNP-IgE (500 ng/ml) in RPMI-1640 medium for at least 2 h before performing assays. For LTC_4_, cells were adjusted to 1 × 10^6^ cells/ml and seeded in 96-well plates. TH was added to a final concentration of 0.1 and 0.2 mM for 1 h before activation with DNP-HSA 100 ng/ml for 15 min. For PGD_2_, cells were seeded in 96-well plates and incubated with TH for 1 h before activation with 100 ng/ml DNP-HSA for 8 h. Supernatants were collected for analysis. Serum from OVA-induced asthma and PSA model mice was collect and analyzed according to the manufacturer’s instructions (Cayman Chemical, MI, United States).

### Quantitative Real-Time PCR

BMMCs were pretreated with DNP-IgE (500 ng/ml) in RPMI-1640 medium for at least 2 h before performing assays. For PCR, cells were adjusted to 1 × 10^6^ cells/ml and seeded in 12-well plates. TH (0.1, 0.2 mM) was added for 1 h, and cells were stimulated with DNP-HSA (100 ng/ml) for 4 h at 37°C and 5% CO_2_ in an incubator. RNA was extracted using TRIzol (TaKaRa, Kusatsu, Shiga, Japan) followed by synthesis of cDNA via reverse transcriptase (TaKaRa, Kusatsu, Shiga, Japan). Quantitative RT-PCR analysis were performed using SYBR Green (TOYOBO, Osaka, JAPAN), and the results were analyzed using the 2−ΔΔCT method and normalized to β-actin expression.

The following primers were used:

Mouse IL-6 forward, 5′-CTG​CAA​GAG​ACT​TCC​ATC​CAG​TT-3′, IL-6 reverse, 5′-GAA​GTA​GGG​AAG​GCC​GTG​G-3′; mouse TNF-α forward, 5′-CGA​GTG​ACA​AGC​CTG​TAG​C-3′, TNF-α reverse, 5′-GGTGTGGGTGAGGAGCACAT-3′; mouse β-actin forward, 5′-TCA​GCA​ATG​CCT​GGG​TAC​AT-3′, mouse β-actin reverse, 5′-ATC​ACT​ATT​GGC​AAC​GAG​CG-3′.

### Western Blot

BMMCs were homogenized, and the protein concentrations of the cell lysates were determined using Bradford reagent (Thermo Scientific, United States). Nuclear and cytoplasmic extractions were prepared as instructed by the manufacturer (Beyotime, Shanghai, China). Cell lysates were denatured for 10 min at 99°C in loading buffer. SDS-PAGE (8–10%) was used to separate samples, which were then transferred to nitrocellulose membranes. Membranes were blocked in 5% nonfat milk diluted in TBS-T for 1 h before incubation with primary antibodies (Cell Signaling Technology, Danvers, MA, United States) at a 1:1000 dilution. Secondary antibodies included horseradish peroxide-conjugated goat anti-rabbit and anti-mouse IgG at a 1:2500 dilution.

### RNA-Sequencing

BMMCs were treated with DNP-HSA for approximately 10 min with or without TH. Procedures for RNA preparation, library construction and sequencing on the BGISEQ-500 platform have been described in detail previously (Xin et al., 2017). The fold changes were also estimated according to the fragments per kilobase of exon per million fragments mapped (FPKM) in each sample. Differential expression analysis was performed using PossionDis with false discovery rate (FDR) ≤ 0.05 and |Log2Ratio| ≥1. KEGG enrichment analysis of annotated differentially expressed genes was performed by Phyper based on the hypergeometric test. We uploaded the raw data of this RNA-seq to the Sequence Read Archive (SRA) database. The sequence data were deposited in the BioSample database under the SRA accession number PRJNA704504.

### Animals

Female BALB/c mice (18–22 g) were purchased from the Shanghai SLAC Laboratory (Shanghai, China) and housed in an SPF (specific pathogen-free) animal room. All animal experiments were designed and implemented in strict accordance with the state’s regulations and the animal center of Shanghai University of Traditional Chinese Medicine. The feeding conditions were as follows: constant temperature (22 ± 1)°C and humidity (55 ± 5%) were maintained in the laminar flow frame of super clean organisms (SPF), 12 h alternating light and dark, and the laboratory and feeding environments were regularly disinfected by ultraviolet light. All experimental operations were performed following the 3R principle to provide humanitarian care. Mice were provided a normal diet and drinking water and adapted to the new environment for at least one week before starting the experiment. For the passive systemic anaphylaxis (PSA) model, mice were randomly divided into 4 groups: normal control group, positive control group, low-dose TH (50 mg/kg) group, and high-dose TH (200 mg/kg) group. Mice were injected with anti-DNP-IgE (2 µg in 100 µl PBS) I.V. and after 24 h, they were given TH or vehicle for 1 h before I V injection with DNP-HSA (2 mg in 200 µl PBS). After 5 min, the mice were anesthetized with 1% pentobarbital sodium. Blood was collected and kept at 4°C for 6 h before centrifugation. Serum was collected for further analysis.

For the OVA-induced asthma model, mice were randomly divided into 5 groups: normal control group, positive control group, low-dose TH (50 mg/kg) group, high-dose TH (200 mg/kg) group, and dexamethasone (DEX) group (0.5 mg/kg). Mice were sensitized by injection with OVA i.p (20 µg in PBS and alum) on day 0 and day 14 in a total volume of 200 µl. On days 15–21, mice were treated with vehicle, TH or DEX p.o. On day 22, 23 and 24 mice were treated with OVA (1% in PBS) aerosolized in an airtight box for 30 mins. On day 25, mice were anesthetized with 1% pentobarbital sodium and then blood collection was performed. Lung and spleen were collected for further analysis.

### Histological Analysis and Immunohistochemistry

After the last OVA challenge, mice were sacrificed and lung tissues were removed and fixed in 4% phosphate-buffered paraformaldehyde, embedded in paraffin, and then stained with hematoxylin and eosin (H&E) and periodic acid Schiff (PAS). The degree of lung inflammation was scored on a subjective scale of 0–4 as previously described (Kim et al., 2019) to evaluate histological damage. For Immunohistochemistry staining, mouse anti-rabbit c-Kit (GB11073, Servicebio, Hubei, China, 1:200 dilution) were used as the primary antibodies and goat anti-rabbit IgG as the secondary antibody (GB23303, Servicebio, Hubei, China, 1:200 dilution). IHC was performed using standard protocols (Leonhardt et al., 2003). Images were captured using DP-72 microscope (Olympus, Tokyo, Japan). The threshold values show the quantification of the integrated OD (IOD) in the different groups using Image Pro-Plus 6.0.

### Statistical Analysis

Data and statistical results are presented as the mean ± S.E.M., and all results were derived from at least 3 independent experiments. Statistical analysis was conducted using GraphPad Prism Software 8.0 (San Diego, CA, United States). Differences between two groups were analyzed using an unpaired Student’s t test, and multiple comparisons were assessed using one-way analysis of variance (ANOVA) with Tukey’s multiple comparison test. *P* < 0.05 was considered a significant difference.

## Results

### TH Inhibited Mast Cell Degranulation and Decreased Inflammatory Cytokines in BMMCs

**FIGURE 1:**
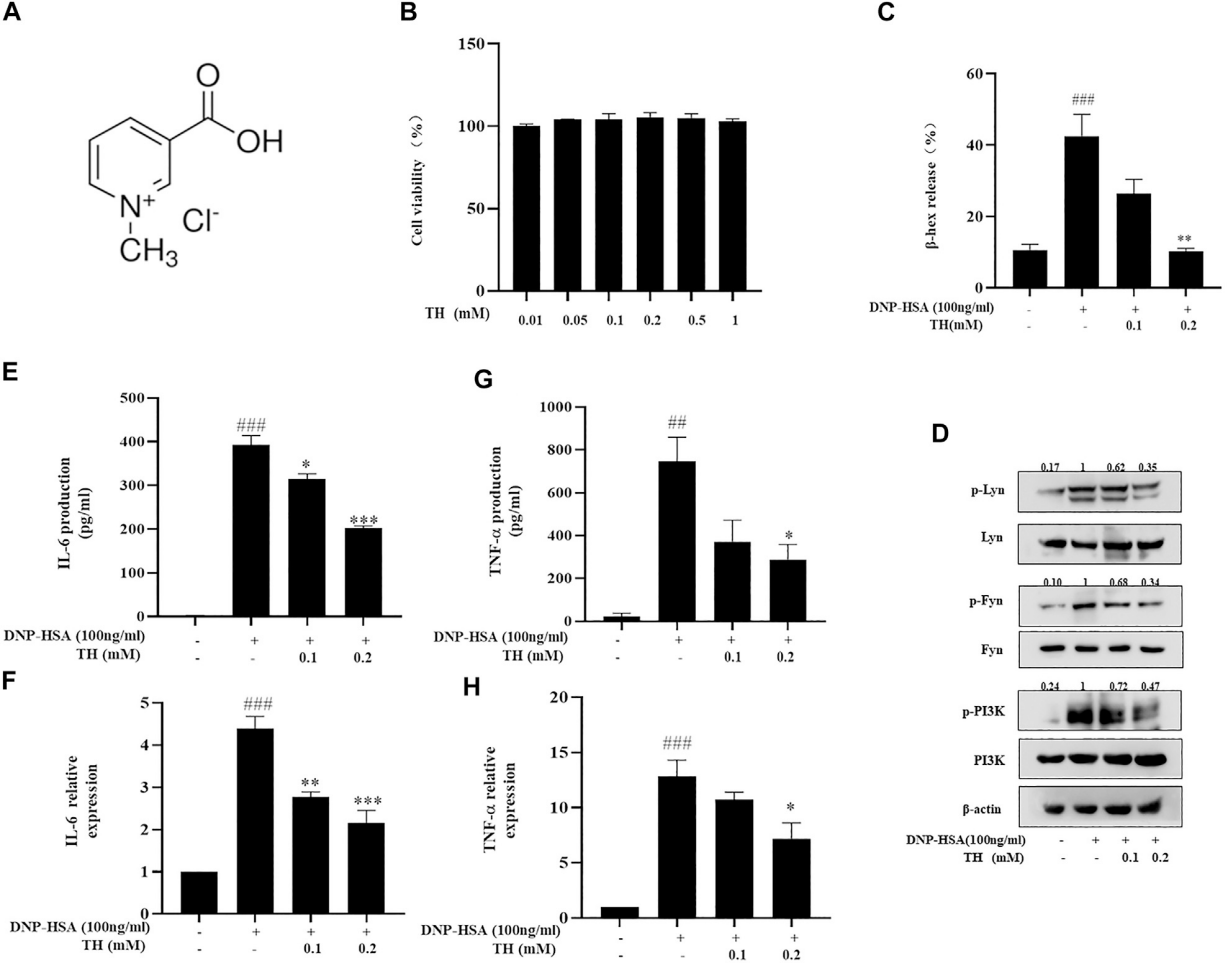
TH inhibited degranulation and decreased cytokine production in BMMCs. **(A)** The chemical structure of TH. **(B)** Cytotoxicity of TH in BMMC. Cells were treated with various concentrations of TH, and cell viability was measured using the MTT assay. BMMCs were incubated with TH and then stimulated with DNP-HSA for different times. Supernatants and cells were collected and analyzed. **(C,D)** β-hex release (at 15 min) and the levels of phosphorylated proteins Fyn, Lyn and PI3K. **(E,F)** IL-6 and TNF-α production determined by ELISA. **(G,H)** IL-6 and TNF-α expression determined by PCR. The data shown are representative of three independent experiments. ^###^ *p* < 0.001 compared to nontreated group, ^*^ *p* < 0.05, **p < 0.01, ***p < 0.001 compared to DNP-HSA.

To detect the antiallergic activity of TH, we chose to investigate mast cells, which play a critical role during the acute stage of allergic reaction. We first examined the cytotoxic effect of TH on BMMCs through MTT assay. BMMCs were treated with various concentrations of TH, and we found no significant cell toxicity at 1 mM (Figure 1B). Because TH showed little toxicity in mast cells, we chose a moderate concentration (0.2 mM) as the highest concentration in subsequent cell experiments. Next, we detected the degranulation of BMMCs after DNP-HSA activation. Mast cells released 40% β-hex compared to the nontreated group. We found that TH significantly reduced the percentage of IgE-induced β-hex release in a dose-dependent manner (Figure 1C). The degranulation process is controlled by early FcεRI-mediated signaling including phosphoinositide 3-kinase(PI3K) family members.TH inhibited the phosphorylation of Lyn, Fyn and PI3K resulting from the aggregation of FcεRI (Figure 1D). The activation of BMMCs also causes the release of cytokines and the production of inflammatory mediators. ELISA kits were used to determine the amounts of cytokines released. The results showed that TH suppressed the secretion of inflammatory cytokines such as TNF-α and IL-6. Quantitative real-time PCR showed the same results (Figure 1E–H). These data preliminarily suggest the inhibitory effect of TH on mast cells.

### TH Suppressed Inflammatory Mediators From Activated Mast Cells *In Vitro* and *In Vivo*

**FIGURE 2:**
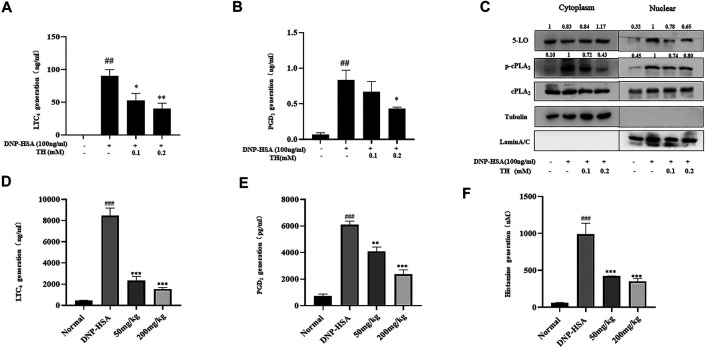
TH suppressed inflammatory mediators *in vitro* and *in* vivo. **(A,B)** LTC_4_ and PGD_2_ production. **(C)** The levels of 5-LO, p-cPLA_2_, cPLA_2_ in cytosol and nucleus were analyzed by western blot. The data shown are representative of three independent experiments. **(D)**The levels of LTC_4_, PGD_2_ and histamine in serum from the PSA model. The data shown are representative of three independent experiments. ^###^ *p* < 0.001 compared to nontreated group, ^*^ *p* < 0.05, **p < 0.01, ***p < 0.001 compared to DNP-HSA.

LTC_4_ and PGD_2_ are two potent mediators related to many allergic diseases. LTC_4_ is generated by arachidonic acid (AA) from membrane phospholipids by cytoplasmic phospholipase (cPLA_2_) and oxygenation of free AA by 5-lipoxygenase (5-LO). The synthesis of PGD_2_ occurs in a biphasic manner, immediate COX-1-dependent and inducible COX-2-dependent de novo PGD2 generation. Our results showed that TH treatment dose-dependently decreased the production of LTC_4_ and PGD_2_ (Figure 2A,B). Western blot analysis showed that the phosphorylation of cPLA2 and nuclear translocation of 5-LO were controlled (Figure 2C). The systemic allergic reactions occur immediately after challenge. Since TH has inhibitory effects against mast cell activation *in vitro*, a passive systemic anaphylaxis (PSA) model was used to investigate the antiallergic effects of TH *in vivo*. Mice were divided into four groups. Blood samples were collected via cardiac puncture, and LTC_4_, PGD_2_ and histamine in serum were analyzed. We found that the amounts of LTC_4_, PGD_2_ and histamine were significantly increased in the vehicle group and decreased in the TH-treated group (Figure 2D–F), which was consistent with the *in vitro* results.

### Inhibitory Effect of TH on the NF-κB and MAPK Signaling Pathways in IgE/Ag-Induced BMMCs

**FIGURE 3:**
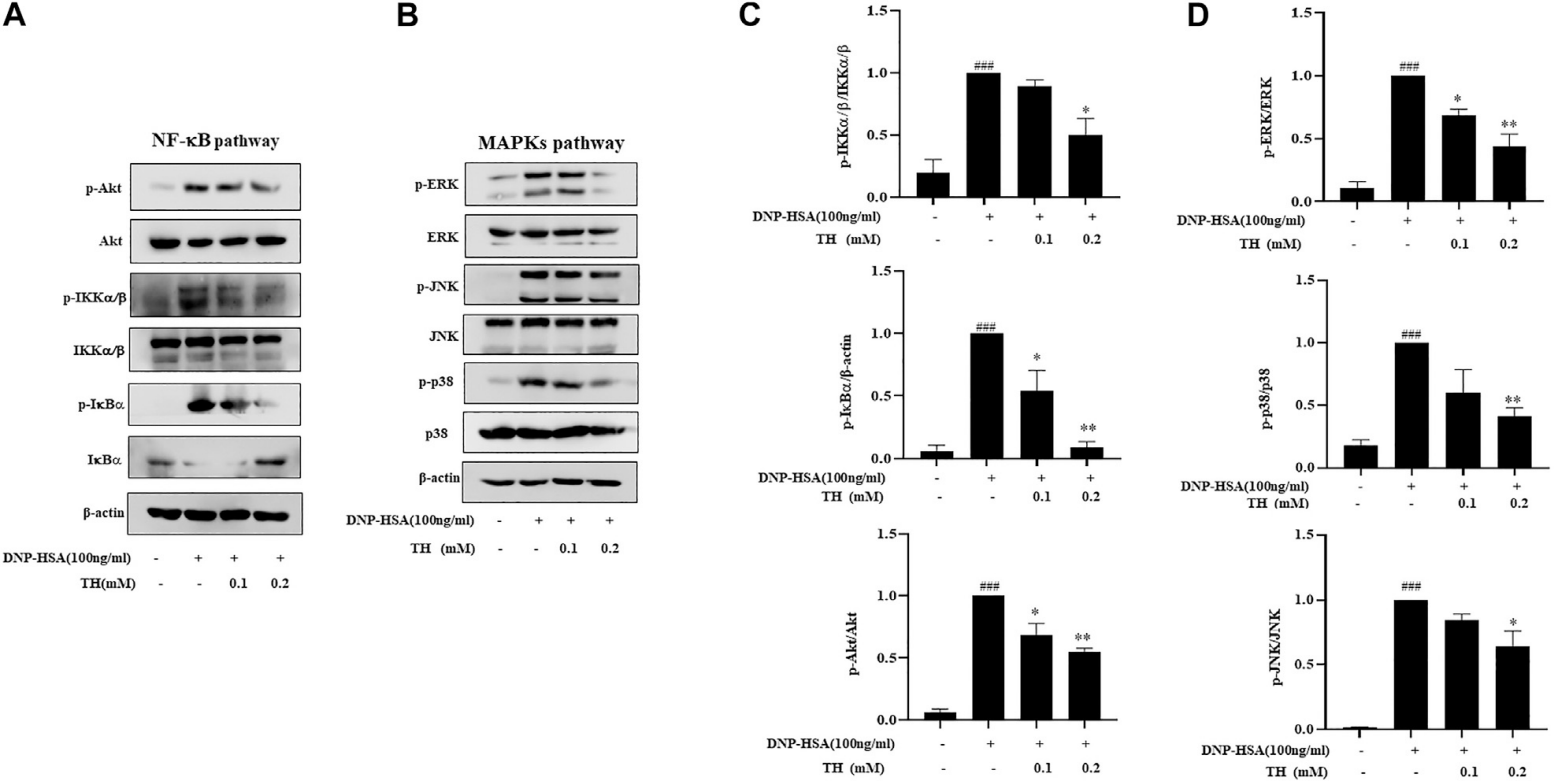
TH inhibited the NF-κB and MAPKs signaling pathways. Cells were pretreated with TH for 1 h and then stimulated with DNP-HSA for 15 min. **(A,B)** The levels of phosphorylated proteins in the NF-κB and MAPK pathways were measured by western blot. **(C,D)** The relative ratios of p-Akt, p-IKK, p-IκBα, p-ERK, p-JNK and p-p38 by measuring the immunoblot band intensities. The data shown are representative of three independent experiments. ^###^ *p* < 0.001 compared to nontreated group, ^*^ *p* < 0.05, **p < 0.01, ***p < 0.001 compared to DNP-HSA.

**FIGURE 4:**
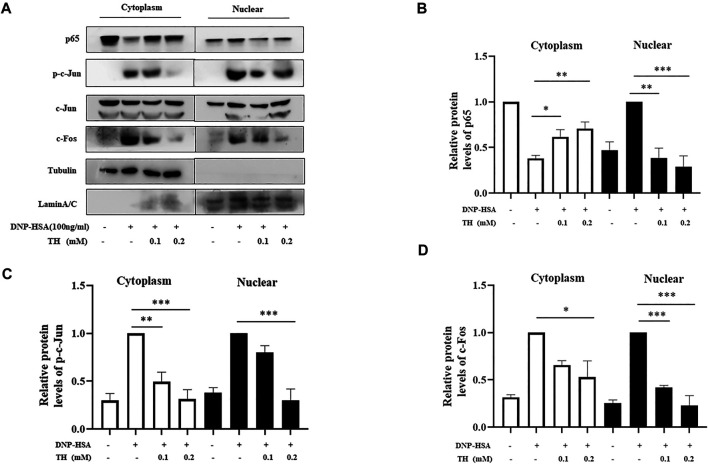
TH inhibited the nuclear translocation of NF-κB, and AP-1. **(A)** Cells were collected after incubation with TH and stimulation with DNP-HSA. The levels of the transcription factors p65, p-c-Jun, c-Jun and c-Fos were determined using western blotting. **(B–D)** The relative ratios of p65, p-c-Jun, c-Jun and c-Fos in cytoplasmic and nuclear extracts. The data shown are representative of three independent experiments. ^*^ *p* < 0.05, **p < 0.01, ***p < 0.001 compared to DNP-HSA.

**FIGURE 5:**
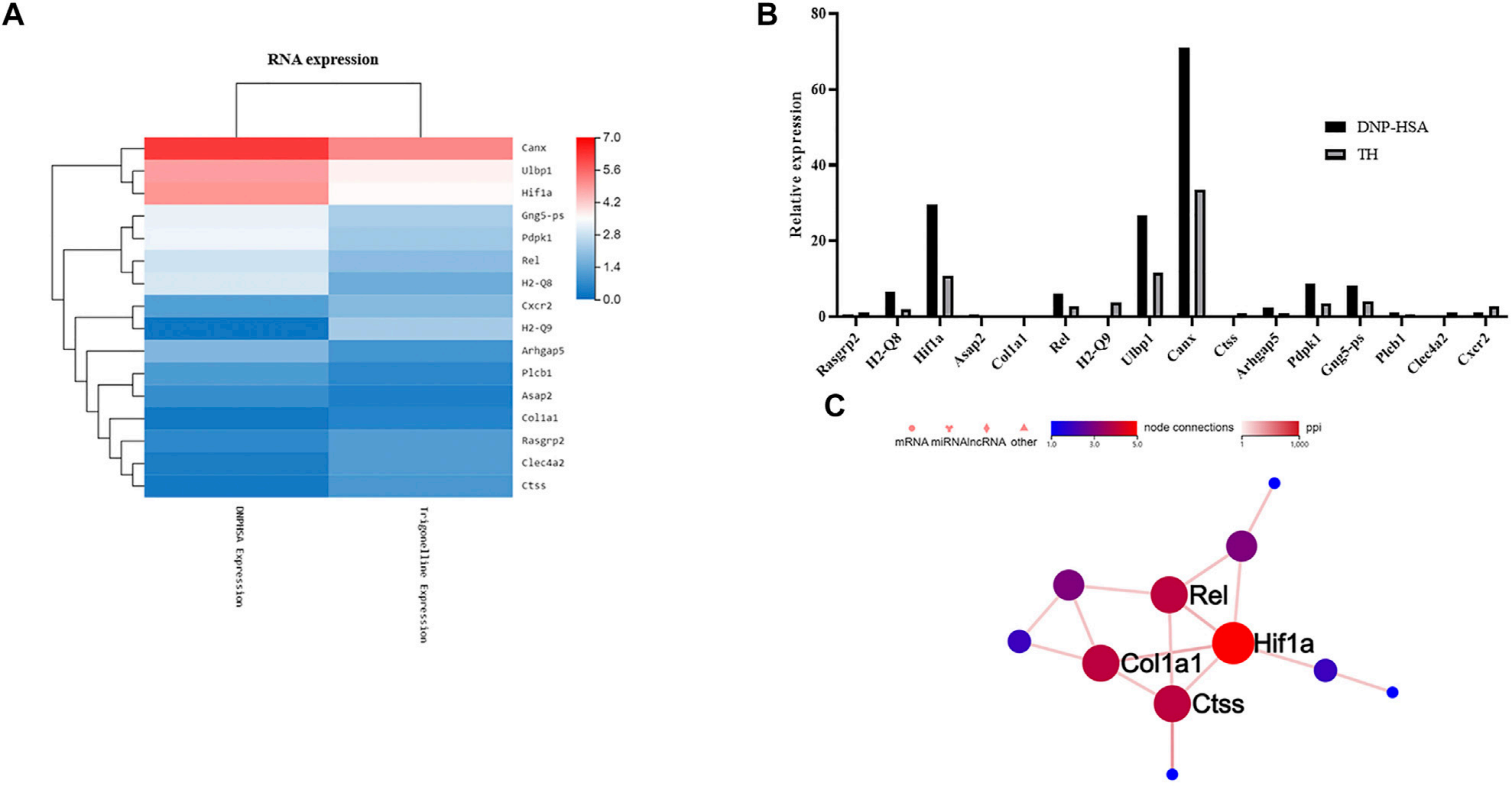
Effect of TH on differentially expressed genes. **(A)** Heatmap of gene expression in TH-treated BMMCs compared to DNP-HSA treated BMMCs. Red and blue represent high- and low- expression levels of the indicated genes respectively. **(B)** Differences in gene expression levels calculated by FDR. **(C)** The protein interactions of these genes. HIF-1α was most related among the differentially expressed genes.

To explore the inhibitory mechanism of TH, we investigated whether TH inhibited NF-κB and MAPKs, two classical downstream signaling pathways in inflammatory responses, in BMMCs. As expected, the phosphorylation of Akt, IKK and IκBα from the NF-κB pathway as well as in MAPK pathways, and the phosphorylation of proteins such as ERK, JNK and p38, were all inhibited (Figure 3). When both signaling pathways are activated, the translocation of the transcription factors NF-κB and AP-1 is an important stage to trigger further events. As they translocate into the nucleus, they bind to DNA and then produce inflammatory mediators. Western blot analysis showed that TH prevented the nuclear translocation of p-65 and AP-1 to some extent (Figure 4). To unravel the potential signaling pathway involved in BMMC-mediated inflammation, we next performed an RNA-sequencing analysis on BMMCs that were cultured for 4 hours in the presence of PBS and TH (0.2 mM). As shown in Figure 5, hypoxia-inducible factor (HIF)-1α displayed an obvious RNA expression difference. Studies have indicated that HIF-1α plays a role in human allergic airway diseases. This gene may be a potential target of the inhibitory mechanism and still needs further exploration and verification.

### TH Attenuated Symptoms and Cytokine Production in the Allergic Asthma Model

**FIGURE 6:**
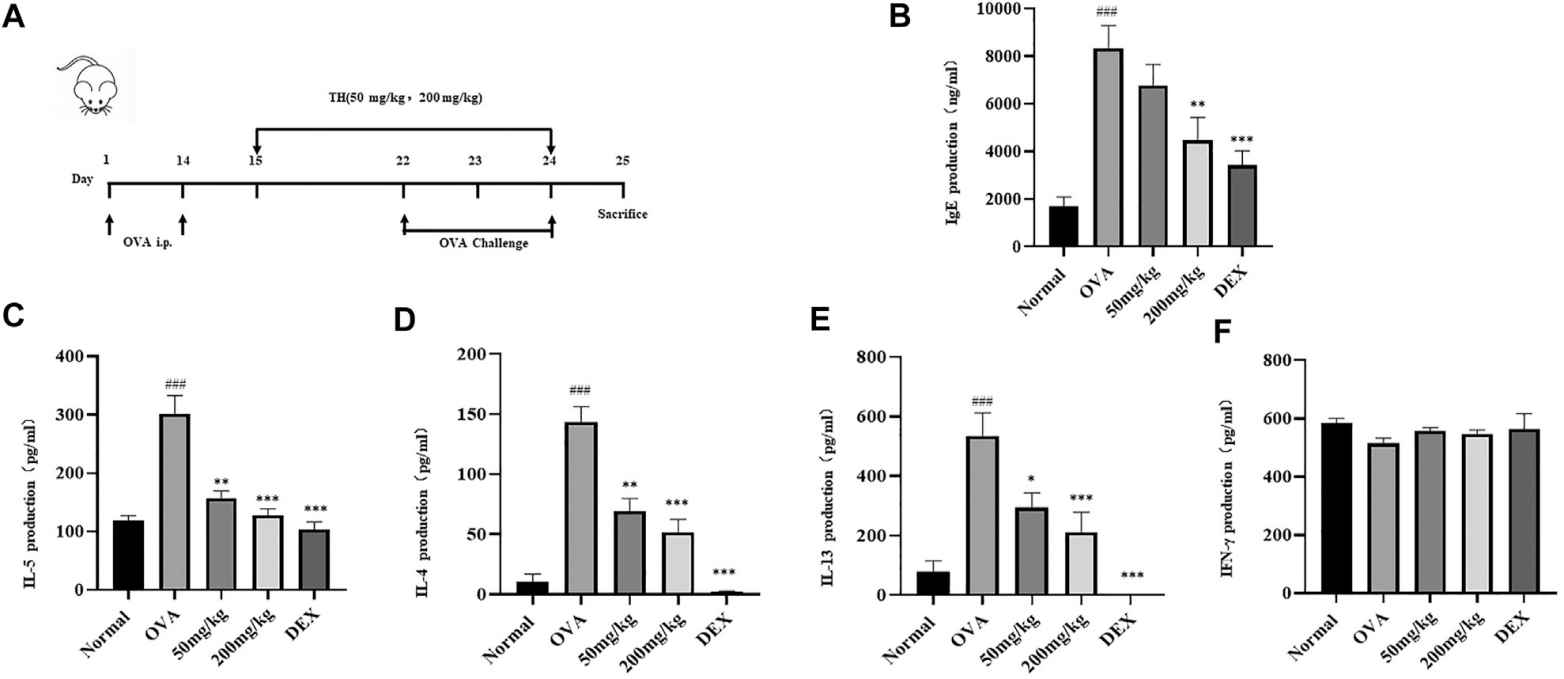
Effect of TH on IgE and Th1/Th2 cytokines in lung tissues. **(A)** The flowchart of the OVA-induced asthma model. Blood samples were collected by cardiac puncture and the level of IgE was determined **(B)**. Lung tissue homogenate was used to determine the levels of IL-5, IL-4, IL-13 and IFN-γ **(C–F)**. The data are presented with *n* = 10 in each group. ^##^ *p* < 0.01, ^###^ *p* < 0.001 compared to the normal group. **p < 0.01, ***p < 0.001 compared to OVA treated group.

**FIGURE 7:**
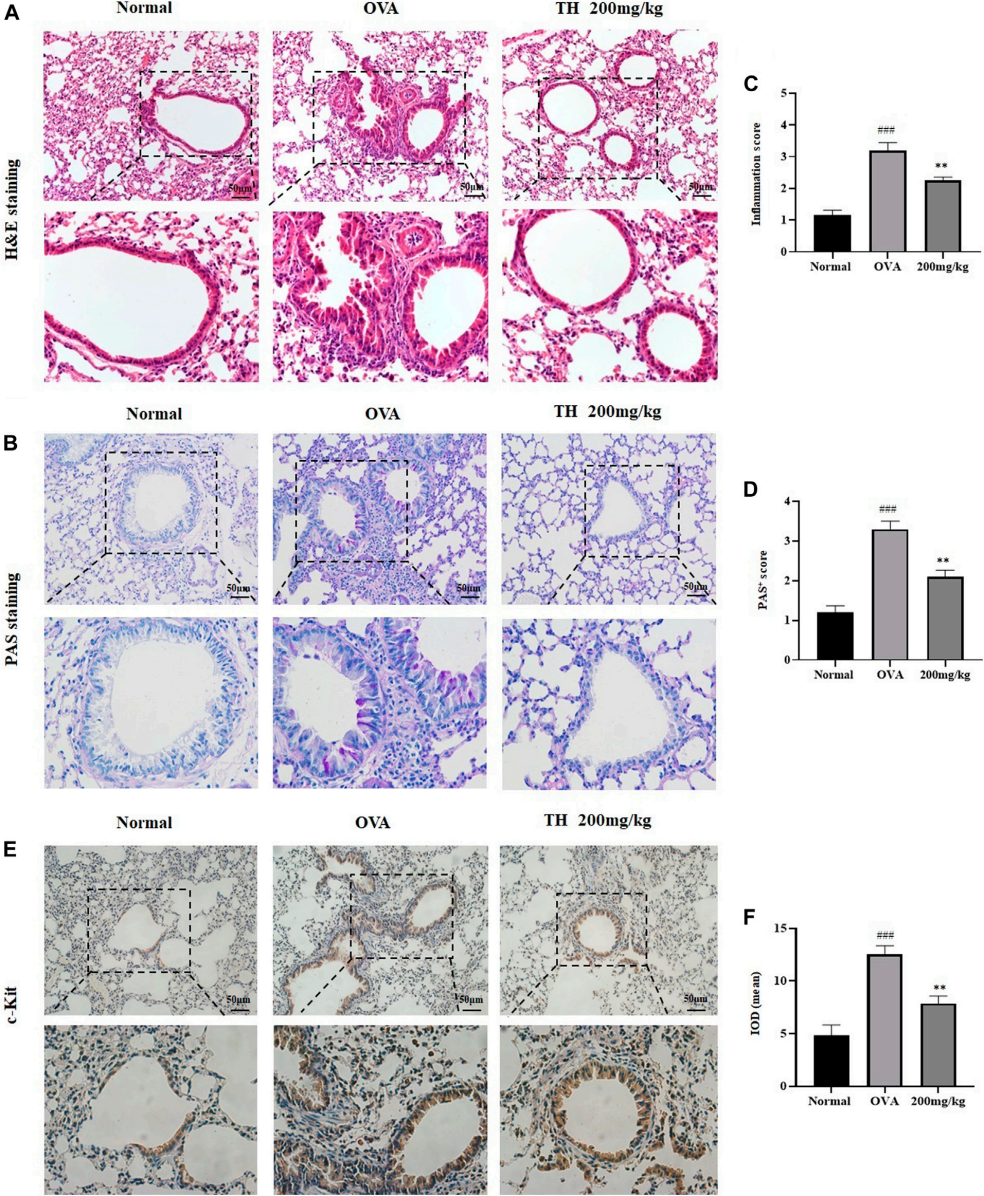
Histology of lung tissue. Histological evaluation. **(A,B)** Representative H&E and PAS-stained sections of the lung and higher magnifications. The infiltration of inflammatory cells and the amount of mucus secretion are shown. The scale labels are 50 μm. **(C,D)** Histological score of inflammatory cell infiltration. IHC staining of c-Kit. **(E,F)** Representative c-Kit stained sections of the lung and higher magnifications. The threshold values show the quantification of the integrated OD (IOD) in the different groups using Image Pro-Plus 6.0. ^###^ *p* < 0.001 compared to the normal group. **p < 0.01, compared to OVA-treated group.

To further clarify the *in vivo* effect of TH, an OVA-induced asthma model was established, and mice were divided into five groups. OVA was injected separately on days 0 and 14, TH was orally administered to the mice for totally 10 times from day 15 to day 24, and then OVA aerosol was administered to induce allergic asthma from day 22 to day 24 (Figure 6A). We measured the serum IgE level, which was decreased in a dose-dependent manner, and the high dosage (200 mg/kg) showed a significant inhibitory effect (Figure 6B). The infiltration of inflammatory cells and the amount of mucus secretion are the important indicators reflecting the degree of asthma symptoms, thus the histology of the lung tissue was evaluated via H&E and PAS staining (Figures 7A–D). Immunohistochemistry of c-kit showed a decrease in mast cell amounts (Figures 7E,F). We found that TH-treated mice showed less inflammatory cell infiltration and mucus secretion in lung tissue than OVA-treated mice. Because asthma is a Th2 cell-related disease, the balance of Th1/Th2 cytokines is crucial. We analyzed Th1/Th2 cytokine levels in lung tissue and found that after treatment with TH, the levels of IL-4, IL-5, and IL-13 all decreased, and the effect of high dose was similar to that of the DEXA group, while the IFN-γ level showed no significant difference compared to that of OVA-treated group. (Figures 6C–F).

### TH Suppressed Inflammatory Signals in Spleens From a Murine Model of Asthma

**FIGURE 8:**
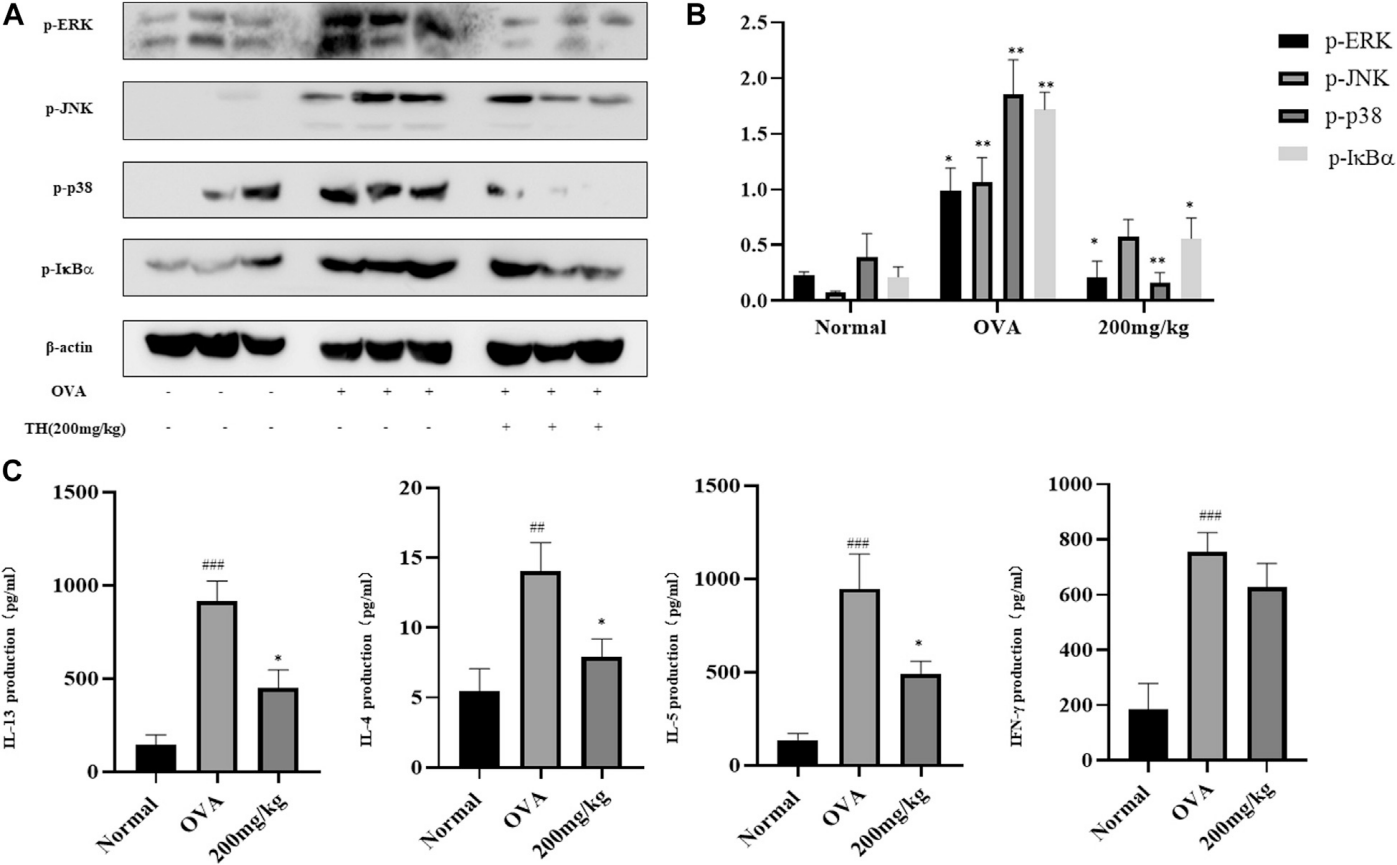
TH suppressed inflammation signals in spleen. Spleen cells were collected after stimulation with OVA (100 μg/ml) for 3 days and were used for western blotting **(A)**. The relative protein level ratios of p-ERK/β-actin, p-JNK/β-actin, p-p38/β-actin, and p-IκBα/β-actin were determined by measuring the immunoblot band intensities **(B)**. Cytokine concentrations were determined by ELISA **(C)**. The data are presented with *n* = 10 in each group. ^#^ *p* < 0.05, ^##^ *p* < 0.01, ^###^ *p* < 0.001 compared to the normal group. **p < 0.01, ***p < 0.001 compared to OVA treated group.

The spleen is generally recognized as an important peripheral immune organ that contains many types of immune cells. We collected spleens from sacrificed mice and stimulated spleen cells with OVA for 3 days. Supernatants were used to detect cytokines by ELISA. As shown in Figure 8C, the levels of IL-4, IL-5, and IL-13 were increased after activation. A high dose of TH could effectively prevent the production of these cytokines but still showed little effect on IFN-γ, which are consistent with the results from lung tissue. Spleen cells were used for western blot analysis. The results showed that a high dose of TH suppressed the phosphorylation of ERK, JNK, p38 related to MAPKs, and IκBα from the NF-κB pathway (Figures 8A,B), indicating that TH could also decrease the activation of the inflammatory signals in splenocytes.

## Discussion

Chinese herbal medicines have been used for thousands of years and have spread worldwide in recent years. Natural products show great potential in treating clinical diseases. *Leonurus japonicus* Houtt. is a traditional Chinese medicine, that has pharmacological activities mainly focused on gynecology and cardiovascular diseases (Miao et al., 2019). Total alkaloids are the active components in Leonurus, the related preparations such as *Leonurus japonicus* capsule and injection have been used in the clinic (Yu et al., 2019). Accumulating evidence shows that Leonurus also has anti-inflammatory functions (Yang et al., 2020). Trigonelline is an alkaloid extracted from *Leonurus japonicus* Houtt., which can also be found in fenugreek seeds and coffee beans. Several pharmacological activities have been reported. As an important component of coffee, trigonelline could effectively inhibit the isolated gut microbes which produce choline metabolism responsible for cardiovascular risk (Anwar et al., 2018; Midttun et al., 2018). Other findings report that trigonelline protects hippocampal neurons from OGD/R-induced injury, indicating its neuroprotective roles in brain pathology (Qiu et al., 2020). However, as a vital alkaloid in Leonurus, the anti-inflammatory effect of trigonelline has not been well studied. Here, we detailed its antiallergic properties both *in vitro* and *in vivo*, which may contribute to exploring new clinical uses for Leonurus.

Mast cells, sentinels of the innate immune system, are important mediator-secreting cells in IgE-mediated type I reactions (Galli and Tsai, 2012). When the antigen is bound to the IgE/FcεRI complex on the cell surface, mast cells are activated and then secrete many allergic mediators including histamine, lipid mediators and cytokines. Following the activation, histamine is rapidly dissociated from the granule matrix by exchange with sodium ions in the extracellular environment (Xie and He, 2005). This dissociation can be used as a marker of mast cell degranulation. LTC_4_, a class of inflammatory lipid mediators, is synthesized from arachidonic acid via 5-LO in mast cells (Kanaoka and Austen, 2019). Inhibiting 5-LO is believed to be the ideal treatment for allergic diseases and asthma. PGD_2_ is another substantial pro-inflammatory lipid mediator downstream of the arachidonic acid/cyclooxygenase (COX) pathway (Rittchen and Heinemann, 2019). In mouse models of asthma and allergic disease, PGD_2_ regulates many hallmark characteristics including airway hyperreactivity, mucus production and Th2 cytokine levels (Joo and Sadikot, 2012; Nakamura et al., 2015). Inflammatory cytokine secretion, such as IL-6 and TNF-α, is commonly regarded as an important indicator that reflects the severity of BMMC activation and OVA-induced allergic asthma. Thus, detecting these mediators can preliminarily screen the antiallergic effect of trigonelline. Our results indicate the inhibitory effect of TH on these inflammatory mediators.

To further elucidate its latent molecular mechanism, the classical signaling pathway was studied. During early signaling events, mast cell degranulation involves cross-talk between the Fyn and Lyn kinases, and the Fyn-PI3K pathway participates in the calcium-independent pathway leading to degranulation (Ogawa et al., 2014; Yi et al., 2018). The results showed that these upstream signaling proteins were suppressed after treatment with TH. NF-κB mediated gene expression regulates many cellular processes. Dysregulation of NF-κB signaling can be responsible for many inflammatory and autoimmune diseases (Ben-Neriah and Karin, 2011). For mast cells, activation of downstream signaling molecules such as Akt can lead to signaling cascades (Nakajima et al., 2019; Kim et al., 2020), eventually culminating in the activation of the IKK complex, and the inhibitory cytoplasmic NF-κB chaperone IκBα, leading to the subsequent phosphorylation of related proteins (Afonina et al., 2017). This phosphorylation results in IκBα ubiquitination, proteasome-mediated degradation and dissociation from NF-κB, leading to the nuclear translocation of NF-κB dimers. MAPKs can regulate the expression of many genes through their action on transcription factors such as NF-κB and AP-1 (Lu et al., 2013). A previous study showed that inhibition of both p38 and ERK decreased p65 phosphorylation, and blunted the upregulation of NF-κB-dependent cytokines, suggesting that the cross-talk between MAPKs and NF-κB contributes to cytokine regulation (Sigala et al., 2011). Our results show that TH plays an anti-inflammatory role by suppressing the related phosphorylated proteins, thus attenuating the MAPK and NF-κB signaling pathways. We also demonstrated that the inhibition of MAPKs suppresses LTC_4_ production by decreasing the phosphorylation and nuclear translocation of 5-LO and cPLA_2_, verifying the previous ELISA results.

To verify the antiallergic activity of TH in *vivo*, two classic animal models were used. Before starting the animal experiments, we pre-tested the drug toxicity to determine the safe dosage. In ten consecutive days, mice were treated with different dosages of TH and then sacrificed. Body weights and organ shape change were observed to judge the toxicity. (Supplementary Figure S4). We first performed a passive systemic anaphylaxis (PSA) model. Histamine is a key vascular mediator that elicits anaphylactic symptoms in mice after challenge. Mast cells largely contribute to IgE-mediated systemic anaphylaxis via histamine release in response to stimulation with IgE and antigens (Ishikawa et al., 2010). The results showed that TH possesses potential anti-inflammatory properties by inhibiting histamine, LTC_4_ and PGD_2_ in serum.

Next, an OVA-induced asthma model was used to further investigate the effect of TH. Asthma, a chronic inflammatory disease, features variable airflow obstruction due to inflammatory responses, mucus production and airway hyperreactivity (Park et al., 2016). The pathogenesis of asthma is complicated, with numerous involved factors, such as cytokines, chemokines, T-cells and inflammatory cells (Shin et al., 2018). The interaction between mast cells and T cells, regarding cellular functionality and immune responses, can be evaluated in both activating and inhibitory regulations (Elieh Ali Komi and Grauwet, 2018). Mast cells, commonly associated with Th2 immediate hypersensitivity reactions, participate in the downregulation of the allergic response by secreting inhibitory cytokines or mediators that shift the Th1/Th2 balance to Th1 (Bulanova and Bulfone-Paus, 2010; Kulinski et al., 2018). The balance between Th1-secretion and Th2-secretion can alter the MC population via several different mechanisms. Asthma is thought to be a Th2 cell-associated inflammatory disease, and Th2-type cytokines, such as IL-4 and IL-13, which can activate B cells to produce allergen-specific IgE, are considered to drive disease pathology in patients (Lu et al., 2016; Elieh Ali Komi and Grauwet, 2018). In contrast to the positive control group, TH significantly suppressed the type II cytokines such as IL-4, IL-5, and IL-13, in the lung tissues. H&E and PAS staining also showed that TH ameliorated pathological changes in the lungs of asthmatic mice. The inhibitory effects were also demonstrated in spleen cells. However, our studies did not support a role for Th1-secretion in controlling asthma, since the amount of IFN-γdid not show a significant change between the positive and TH-treated groups. This finding may indicate that TH could be used as a type II cytokine-blocking drug but will not affect the production of type I cytokines.

RNA-sequencing analysis showed that TH significantly upregulated 105 genes and downregulated 201 genes (with a P value of less than 0.001) when compared with BMMCs treated with PBS (Supplementary Figure S1). We investigated the most upregulated or downregulated genes in TH versus PBS conditions in the presence of DNP-HSA and selected immune-related genes among 306 genes. HIF-1α is related to immune system disease and displayed an obvious RNA expression difference. Allergen exposure could cause upregulation of HIF-1α and vascular endothelial growth factor (VEGF) in patients with asthma and rhinitis (Huerta-Yepez et al., 2011). Inhibition of PI3K/Akt activity and subsequent blockade of the mTOR-HIF-1α-VEGF module can attenuate typical asthmatic attack in a murine model (Choi et al., 2013). For primary human mast cells, alleviating HIF-1α activation and VEGF production could suppress inflammatory reactions (Nam et al., 2017). The citrus unshiu peel, which has been used traditionally as a medicine to improve bronchial and asthmatic conditions, was verified as an inhibitor of HIF-1α on the HMC-mediated inflammatory responses (Choi et al., 2007).For BMMCs, inflammation can be suppressed via an HIF-1α–dependent blockade of miR-155-5p (Abebayehu et al., 2016). We found there is still a lack of enough research that can directly explain the association between BMMCs and HIF-1α. This needs to be further investigated in future studies.

Since the allergic asthma has intimate links with HIF-1α, further investigation can be focused on the interaction between TH and T cells, and how HIF-1α functions during the BMMC-mediated inflammation process.

In summary, TH was shown to exert the anti-inflammatory effect on mast cell-dependent allergies both *in vitro* and *in vivo*, which could provide a novel therapeutic application for *Leonurus japonicus* Houtt.

## Data Availability

The datasets presented in this study can be found in online repositories. The names of the repository/repositories and accession number(s) can be found in the article/Supplementary Material.

## Abbreviations

AP-1, activator protein 1; Akt, protein kinase B; ERK, extracellular signal regulated kinase1/2; HSA, human serum albumin; IFN-γ, interferon gamma; IKK, I kappa B kinase; IL-6, interleukin-6; JNK, c-jun N-terminal kinase; PBS, phosphate buffer saline; TNF-α, tumor necrosis factor alpha.
