# Supplementary material for: Trigonelline, An Alkaloid From Leonurus japonicus Houtt., Suppresses Mast Cell Activation and OVA-Induced Allergic Asthma
Source: Front Pharmacol. 2021 Aug 4;12:687970. doi: 10.3389/fphar.2021.687970 (PMC8371462; doi:10.3389/fphar.2021.687970)
Supplement: Supplementary file 1 [file DataSheet1.docx]

Supplementary Material

## Supplementary Figures


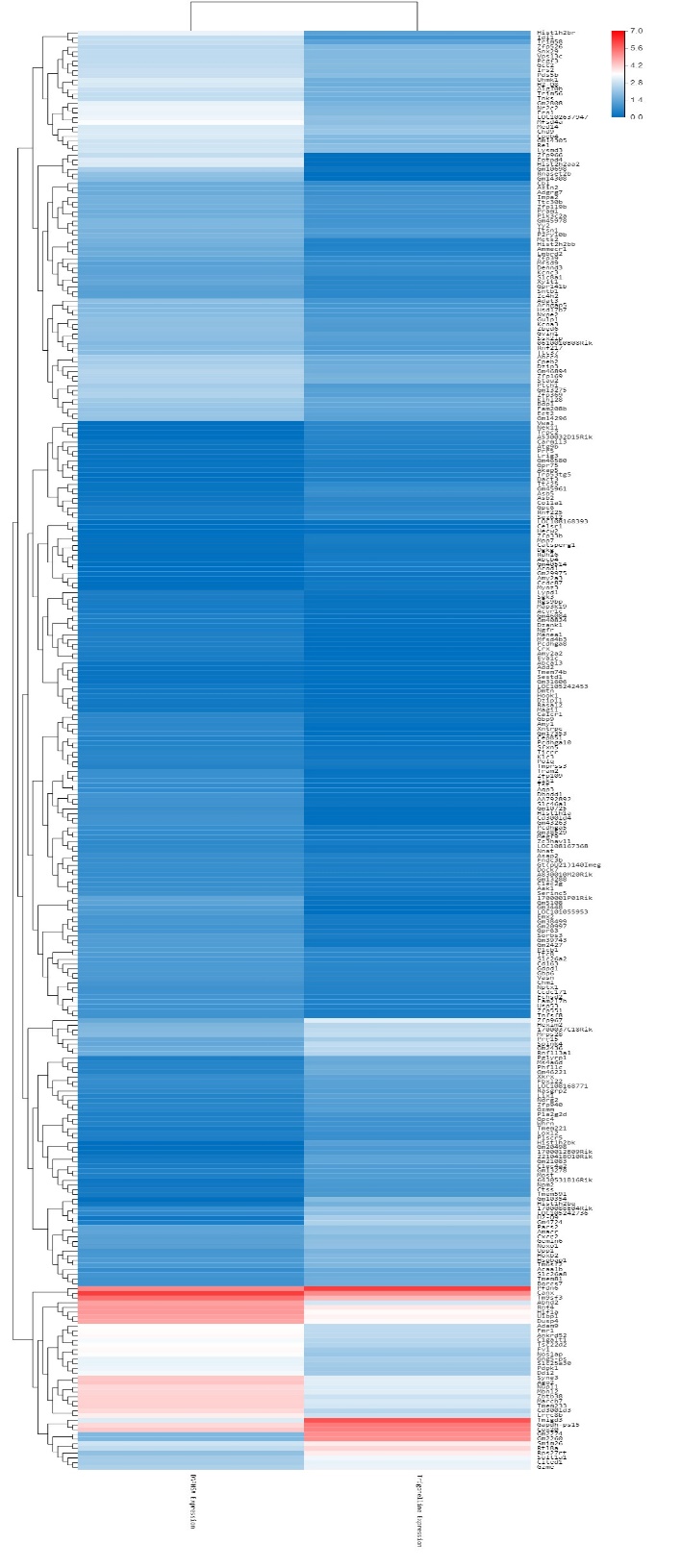


**Supplementary Figure 1.** Heatmap of 306 genes expression of trigonelline treated BMMC on representative differentially expressed genes comparing to DNP-HSA treated. False Dicovery Rate (FDR) ≤ 0.05 and |Log2Ratio| ≥ 1.


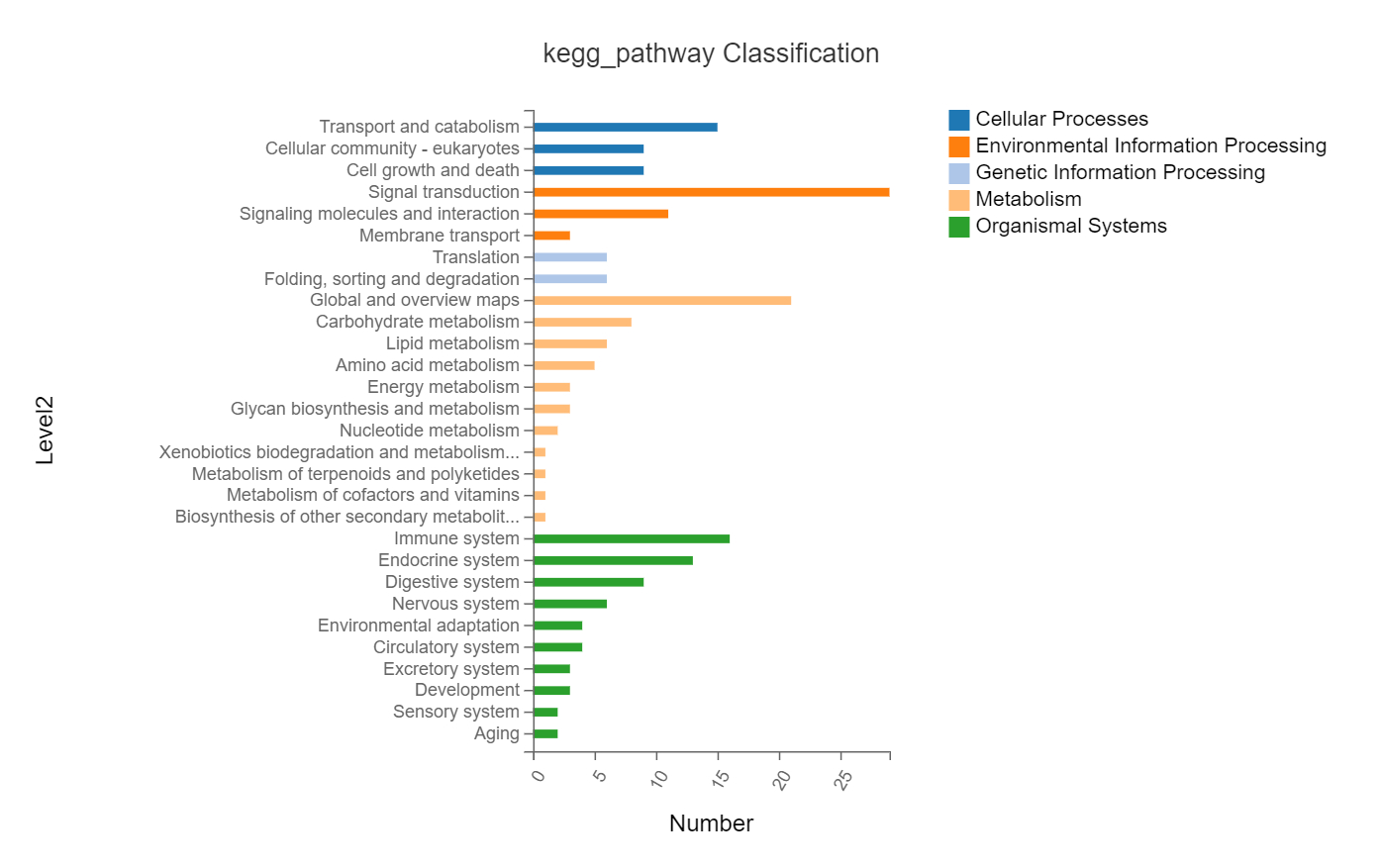


**Supplementary Figure 2.** KEGG pathway database analysis showed the effect of trigonelline related to different kinds of physiological processes.


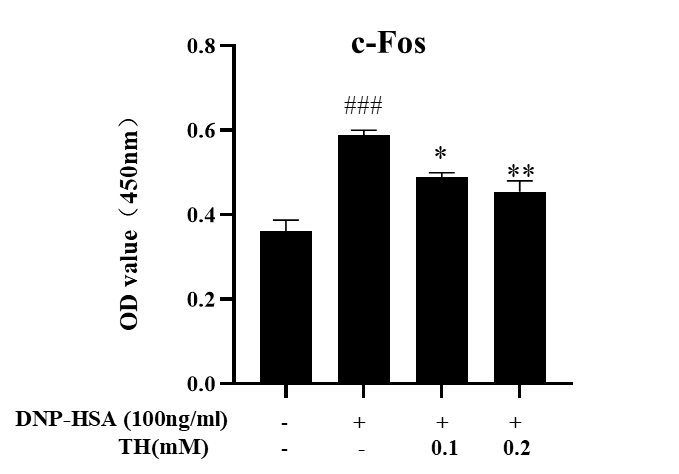

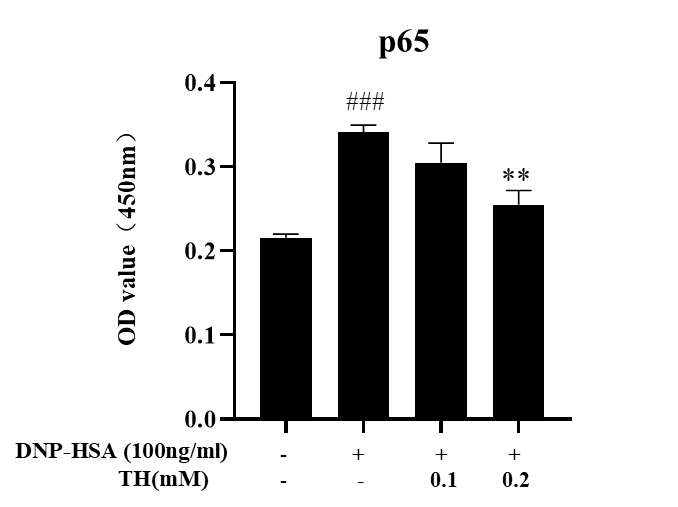


**Supplementary Figure 3.** Nuclear transcriptional factors binding activity of p65 and c-Fos. Results were determined by TransAM ELISA kit.


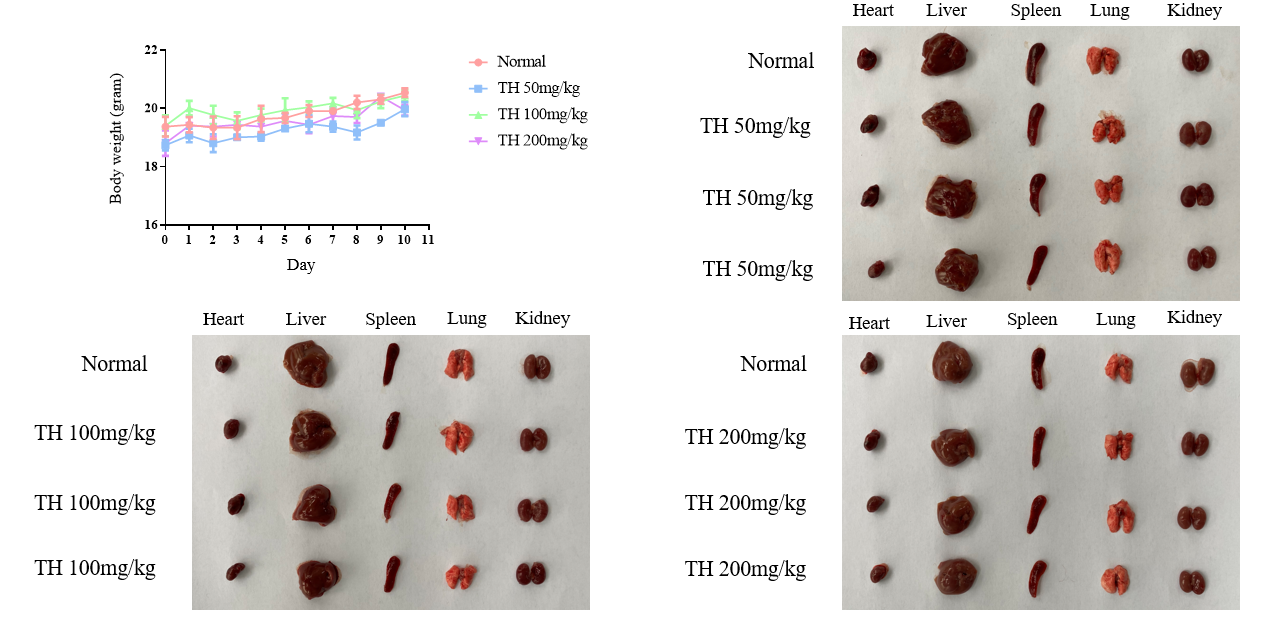


**Supplementary Figure 4.** Body weights and organs from mice in drug toxicity pre-test

















nuclear

cytoplasm

**Supplementary Figure 5.**  p-p65 bands from three independent experiments
